# Quantification and Verification of Cardiorespiratory Fitness in Adults with Prehypertension

**DOI:** 10.3390/sports9010009

**Published:** 2021-01-11

**Authors:** Dharini M. Bhammar, Lung-Chang Chien

**Affiliations:** 1 Department of Kinesiology and Nutrition Sciences, School of Integrated Health Sciences, University of Nevada Las Vegas, Las Vegas, NV 89154, USA; 2 Department of Epidemiology and Biostatistics, School of Public Health, University of Nevada Las Vegas, Las Vegas, NV 89154, USA; Lung-Chang.Chien@unlv.edu

**Keywords:** blood pressure, aerobic fitness, 3-min all-out exercise test, VO_2max_, deconditioning

## Abstract

Background: Low cardiorespiratory fitness is associated with increased risk of hypertension and atherosclerosis in adults with prehypertension. The purpose of this study was to quantify cardiorespiratory fitness and to examine the utility of supramaximal constant-load verification testing for validating maximal oxygen uptake (VO_2max_) attainment in adults with prehypertension. Methods: Eleven adults (four women) with prehypertension (22.5 ± 2.9 y; body mass index (BMI): 24.6 ± 3.2 kg·m^2^) underwent an incremental exercise test followed 15 min later by a verification test at 105% of maximal work rate on a cycle ergometer. Results: There was no statistical difference in VO_2_ between the incremental (2.23 ± 0.54 L·min^−1^) and verification tests (2.28 ± 0.54 L·min^−1^; *p* = 0.180). Only three out of eleven participants had a higher VO_2_ during the verification when compared with the incremental test. If the verification test had not been conducted, one participant would have been incorrectly classified as having low cardiorespiratory fitness based on incremental test results alone. Conclusions: Verification testing validates the attainment of VO_2max_ and can potentially reduce the over-diagnosis of functional impairment (i.e., deconditioning) in adults with prehypertension.

## 1. Introduction

Prehypertension affects one in three adults in the United States [1] and is associated with an increased risk of hypertension [2] as well as cardiovascular morbidity and mortality [3]. Low cardiorespiratory fitness in adults with prehypertension is associated with an increased risk of hypertension [4] and atherosclerosis [5]. Previous studies that have examined the associations between cardiorespiratory fitness and health outcomes in adults with prehypertension have typically quantified cardiorespiratory fitness relative to body weight (i.e., as mL·min^−1^·kg^−1^) or metabolic equivalents (MET) or treadmill test duration, and they have used treadmill as the mode of exercise for fitness assessments [4,6,7,8,9,10]. While treadmill testing per se may not affect maximal oxygen uptake (VO_2max_) levels, the use of exercise test duration from a treadmill protocol as a surrogate for VO_2max_ can negatively influence performance in individuals who carry excess body weight [11]. These traditional methods of quantifying cardiorespiratory fitness could inaccurately underestimate fitness levels in individuals with overweight or obesity, who are also at increased risk of high blood pressure [12], and can confound the associations between cardiorespiratory fitness and negative health outcomes [13]. Both the American Thoracic Society (ATS) [14] and the American Heart Association (AHA) [15] recommend quantifying cardiorespiratory fitness as a percent of predicted VO_2max_, where predicted VO_2max_ is calculated based on ideal body weight. This approach can reduce the underestimation of cardiorespiratory fitness in overweight and obese individuals [16].

Cardiopulmonary exercise testing provides an assessment of VO_2max_ or the highest amount of oxygen that can be delivered by the cardiorespiratory system and utilized by the skeletal system. Hill and Lupton first suggested in 1923 that VO_2max_ represented a point during a graded exercise test where VO_2_ failed to increase despite an increase in work rate (WR) [17]. Since then, the demonstration of a VO_2_ plateau has become an essential marker for the achievement of VO_2max_. Unfortunately, over 40 % of individuals do not achieve a VO_2_ plateau during a graded exercise test despite achieving VO_2max_ [18]. To overcome this limitation, verification testing has been increasingly recommended for validating the achievement of VO_2max_ from an incremental or graded exercise test in lieu of a VO_2_ plateau [18]. However, the verification testing approach has not been previously evaluated in adults with prehypertension, who could benefit from the accurate measurements of cardiorespiratory fitness and consequently the accurate prediction of chronic disease risk.

The purpose of this study was to quantify cardiorespiratory fitness based as a percent of predicted VO_2max_ and to examine whether a supramaximal constant-load verification test validates the attainment of VO_2max_ from an incremental test in adults with prehypertension. Based on published data in recreationally trained adults [19] and adults with obesity [20], we hypothesized that there would be no difference between incremental and verification test VO_2_ in adults with prehypertension.

## 2. Materials and Methods

The Valdosta State University Institutional Review Board approved this study (Protocol number: IRB-03344-2016), and all participants provided written, informed consent. All participants were 18–30-year-old, healthy, non-smoking adults with a body mass index (BMI) between 20–30 kg·m^−2^. Participants with cardiovascular, renal, or metabolic disease were excluded [21]. Height and weight were measured using calibrated scales. Resting blood pressure (BP) was measured once in each arm to confirm a difference of <10 mm Hg between arms. Thereafter, three BP measurements were taken in the left arm on two separate days using an automated monitor (SunTech Tango M2 stress test monitor, SunTech Medical, Morrisville, NC, USA) [22]. All participants had a resting systolic BP between 120–139 or a resting diastolic BP between 80–89 mm Hg, which met the definition of “prehypertension” at the time of study design [22]. Participants were asked to avoid caffeine and exercise for 24 h and food for 3 h prior to all study visits.

### 2.1. Incremental Test 

The incremental test was performed on an electronically braked cycle ergometer (Ergoline VIAsprint 150P, Bitz, Germany). Participants completed a two-min warm-up at 30 Watt (W) for women and 40 W for men. WR was increased in the third minute to 40 W for women and 50 W for men. Thereafter, WR was increased by 20 W for women and 25 W for men every minute until volitional exhaustion. Participants were asked to maintain cadence between 60–70 rpm, but were allowed to increase cadence at higher WR, although they were not allowed to exceed a cadence of 100 rpm. The test was stopped when participants were unable to maintain a cadence of 50 rpm for five consecutive seconds despite encouragement.

### 2.2. Verification Test 

The verification test was performed at least 15 min after the incremental exercise test. The verification test was completed at 105% of the maximal WR from the incremental test until volitional exhaustion. The participants warmed up by cycling for 2 min at 30 W for women or 40 W for men. WR was increased to 105% maximal WR immediately after the warm-up, and participants were encouraged to pedal at a cadence of their choice between 60–100 rpm for as long as possible. The test was stopped when participants were unable to maintain a cadence of 50 rpm for five seconds despite encouragement.

### 2.3. Measurements 

Participants were fitted with an oronasal mask connected to a standard non-rebreathing valve (Hans Rudolph, Shawnee, KS, USA). Minute ventilation (V_E_) and gas exchange (VO_2_ and carbon dioxide production, VCO_2_) were measured with a metabolic measurement system and reported as 20-s averages (TrueOne 2400, Parvo Medics, Sandy, UT, USA). A standard three-point calibration was performed before each test or every 4 h per manufacturer recommendations. Heart rate (HR) was measured continuously (Polar Electro Inc., Bethpage, NY, USA). Ratings of perceived breathlessness (RPB; Borg 0–10 scale) and ratings of perceived exertion (RPE; Borg 6–20 scale) were assessed every min [23]. The highest VO_2_ from the incremental or verification test was accepted as VO_2max_.

### 2.4. Data Management

Data from each test were processed as explained below:

1. Attainment of VO_2_ plateau during the incremental test: For each incremental test, an assessment was made regarding the achievement of a VO_2_ plateau [24,25]. First, we completed a regression of the VO_2_ and WR relationship after excluding the first and last minute of data. Next, we calculated the “expected” increase in VO_2_ from the penultimate to the final stage using the VO_2_/WR regression. Finally, achievement of VO_2_ plateau was accepted when the difference between measured VO_2_ between the penultimate and final stage was less than 50% of the “expected” increase.
2. Difference in VO_2_ between incremental and verification tests: To evaluate whether the highest VO_2_ during the verification test truly exceeded the highest incremental VO_2_, we calculated the “expected” verification VO_2_ at 105% of maximum WR using the VO_2_/WR regression from the incremental test as stated above. If the measured verification VO_2_ was equal to or exceeded the “expected” verification VO_2_, we concluded that the participant achieved a higher VO_2_ during the verification test.
3. Time taken to reach highest VO_2_ during the verification test: Segmental regression analyses were conducted in Prism 8 (version 8.4.2, GraphPad Software, San Diego, CA, USA) to estimate the time at which the VO_2_ reached a “plateau” during the verification test. A similar assessment was conducted in Microsoft Excel (version 2011, Microsoft, Redmond, WA, USA) using visual inspection of the VO_2_ (L·min^–1^) versus time (s) plot to compare the two methods (i.e., Prism 8 vs. visual inspection in Excel).
4. Quantification of cardiorespiratory fitness: VO_2max_ was predicted for each participant using equations from Hansen and Wasserman, with age of 30 years used for adults younger than 30 years [16]:

*Men*: 

Ideal weight (kg) = 0.79 × Height (cm) − 60.7

If actual weight equaled or exceeded ideal weight:

VO_2max_ = 0.0337 × Height (cm) − 0.000165 × Age × Height (cm) − 1.963 + 0.006 × Weight (actual − ideal)

If actual weight was less than ideal weight:

VO_2max_ = 0.0337 × Height (cm) − 0.000165 × Age × Height (cm) − 1.963 + 0.014 × Weight (actual − ideal)

*Women*: 

Ideal weight (kg) = 0.65 × Height (cm) − 42.8

VO_2max_ = 0.001 × Height (cm) × (14.783 − 0.11 × Age) + 0.006 × Weight (actual − ideal)

Percent predicted VO_2max_ was calculated as measured/predicted × 100. For % predicted VO_2max_, >84% was considered normal based on ATS recommendations [14].

### 2.5. Data Analyses 

With an effect size of 1.069 based on previously published data [25] and an alpha of 0.05, a sample size of 11 participants would be able to detect a difference between incremental and verification VO_2_ using a Wilcoxon signed-rank test with a power of 0.87. Mann–Whitney U tests were used to compare sex differences. Wilcoxon signed-rank tests were used to compare differences in variables between maximal and verification tests. Fisher’s exact test was used to examine proportional differences between sexes. Kendall’s tau-b *(τb)* was used to examine associations between variables. Analyses were completed with SPSS software (version 27, IBM, Armonk, NY, USA). In this study, *p* < 0.05 was considered statistically significant.

## 3. Results

Participant characteristics are shown in Table 1. Six participants were normal weight (BMI 18.5–24.9 kg·m^−2^) and five were overweight (BMI 25–29.9 kg·m^−2^). Women had higher diastolic blood pressure when compared with men (Table 1). WR_max_ was higher in men when compared with women (Table 1).

### 3.1. Incremental Test

Seven participants (64%) achieved a VO_2_ plateau during the incremental exercise test (Table 2). Three out of four women did not achieve a VO_2_ plateau during the incremental test, although their peak HR and respiratory exchange ratio (RER) were high, which indicated that they were approaching exhaustion (Table 2). One out of seven men did not achieve a VO_2_ plateau. This participant had the highest BMI in the group, and his HR and RER responses indicate that he may have experienced fatigue prior to achieving VO_2max_ (Table 2).

### 3.2. Verification Test

The verification test duration for participants in this study was 127 ± 20 s (see individual responses in Table 2). Estimates for time taken to reach a plateau during the verification test from Prism 8 and visual inspection in Excel were not statistically different (59 ± 13 and 60 ± 13 s; *p* = 0.929; Wilcoxon signed-rank test), suggesting that both methods can be used to determine the presence of a VO_2_ plateau in a verification test. The minimum duration of the verification test in this study was 96 s, which was above the maximum time taken to attain a VO_2_ plateau from both Prism 8 (72 s) and visual inspection methods (80 s). VO_2max_ (L·min^−1^) was significantly correlated with verification test duration (τb = 0.636, *p* = 0.006). The difference in VO_2_ between incremental and verification tests was not significantly correlated with the verification test duration (τb = 0.309, *p* = 0.186).

### 3.3. Incremental vs. Verification Test

Only three out of eleven participants (27%) had a higher VO_2_ during the verification test when compared with the incremental test based on the “expected” VO_2_ from the VO_2_/WR regression. For one participant, the difference between incremental and verification test VO_2_ was 144 mL·min^−1^, despite achieving a VO_2_ plateau during the incremental test (Table 2). However, the other two participants did not achieve a VO_2_ plateau during the incremental test and had differences of 213 mL·min^−1^ and 252 mL·min^−1^ between the incremental and verification test VO_2_.

On average, respiratory exchange ratio (RER), calculated as VCO_2_/VO_2_, was higher during the incremental when compared with the verification test (Table 3). RER increases during high-intensity exercise due to bicarbonate buffering of hydrogen ions (i.e., metabolic acidosis), which increases the rate of CO_2_ production out of proportion to O_2_ consumption [16]. There were no statistically significant differences for VO_2_, VCO_2_, V_E_, HR, or breathing pattern between the incremental and verification tests (Table 3).

### 3.4. Quantification of Cardiorespiratory Fitness

Using the highest VO_2_ (% predicted) from either the incremental or verification test, we found that two out of eleven participants had below normal levels of cardiorespiratory fitness (i.e., VO_2max_ ≤ 84% predicted; Table 2). If the verification test had not been conducted, one more participant would have been incorrectly classified as having below normal cardiorespiratory fitness (i.e., VO_2max_ ≤ 84% predicted) based on incremental test results alone (Table 2). This participant had not achieved a VO_2_ plateau during the incremental test.

### 3.5. Predictors of Maximal Oxygen Uptake

**Figure 1:**
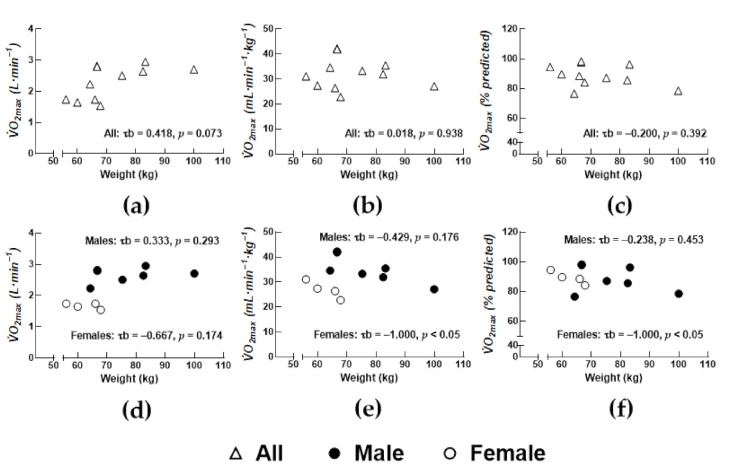
Relationship between total body mass and (**a**) absolute maximal oxygen uptake (VO_2max_), (**b**) relative VO_2max_, and (**c**) VO_2max_ quantified as % predicted for all study participants. Relationship between total body mass and (**d**) absolute VO_2max_, (**e**) relative VO_2max_, and (**f**) VO_2max_ quantified as % predicted for women and men. τb: Kendall’s tau-b.

Total body mass tended to be associated with absolute VO_2max_ in all participants (Figure 1; *p* = 0.073). There were no significant associations between cardiorespiratory fitness (i.e., VO_2max_ quantified as % predicted) and age, anthropometrics, or blood pressure in the total study sample. Cardiorespiratory fitness was inversely associated with total body mass in women (Figure 1f).

## 4. Discussion

A two-step protocol that includes both incremental and verification testing for the measurement of VO_2max_ can confirm the attainment of VO_2max_ in adults with prehypertension. Relying on the incremental test alone could underestimate true VO_2max_ in ≈27% of individuals and could result in misdiagnosing individuals with prehypertension as “deconditioned”. From a methodological standpoint, 80 s represented the longest duration taken for participants to reach a VO_2_ plateau during a supramaximal constant-load verification test completed at 105% of WR_max_.

Accurate assessments of VO_2max_ are critical to risk prediction models in epidemiology as well as for assessing changes in cardiorespiratory fitness after exercise interventions. Therefore, studies in relatively healthy populations who are not at risk of a “symptom-limited” exercise test [26] should consider a two-step protocol that can be completed during a single visit for confirming VO_2max_. In this study, a two-step protocol was successfully completed in 11 young adults with prehypertension. We reported a higher verification VO_2_ when compared with incremental VO_2_ in three out of eleven participants. 

To determine if there is a meaningful difference in VO_2_ between incremental and verification tests, researchers must consider VO_2_ measurement error, which is estimated at 40 mL·min^−1^ for the Parvo Medics TrueOne 2400 [27], and also consider the possibility that a participant may have terminated the incremental test prior to achieving VO_2max_. The present study predicted the expected VO_2_ at maximal and supramaximal WRs from the individual participants’ VO_2_/WR regression and then compared the measured difference between incremental and verification VO_2_ against the predicted difference. Had this approach not been used in the present study, seven out of eleven participants would have been identified with having a higher verification when compared with incremental VO_2_. For four of these seven participants, the difference between incremental and verification tests ranged from 2 to 73 mL·min^−1^. Alternatively, Midgley et al. [28] considered a ≤2% difference between incremental and verification VO_2_ as a criterion for verifying VO_2max_ based on the measurement error in determining VO_2_. Had the present study used the 2% criterion, one additional participant with the 73 mL·min^−1^ difference (≈4%) would have had a higher verification VO_2_ when compared with the incremental VO_2_.

Four out of eleven participants, three of whom were women, did not achieve a VO_2_ plateau during the incremental test in this study. This is consistent with other reports that have demonstrated a failure to achieve a VO_2_ plateau even when true VO_2max_ is achieved in healthy men [24,29,30]. Day et al. [24] noted the absence of a VO_2_ plateau during an incremental test in 83% of 71 men. This was similar to results by Rossiter et al. [29] in a smaller sample, where five out of seven men demonstrated no plateau (i.e., 71.4%). Poole et al. [30] noted the absence of a VO_2_ plateau in three out of eight male subjects (i.e., 38.5%). Sidney and Shephard [31] reported that 79% of men and 75% of women, ages 60–83 years, who gave a good effort during the incremental test, achieved a VO_2_ plateau (defined as a ≤2mL·min^−1^·kg^−1^ increase in VO_2_ with an increase in WR). Based on the very limited sample of women in the present study, it is difficult to speculate if sex differences exist with respect to VO_2_ plateau achievement in prehypertensive adults. However, it should be noted that the original concept of the VO_2_ plateau was derived from multiple constant-load tests and a plateau was accepted when VO_2_ did not differ between two consecutive tests with differing WR [24]. Therefore, a VO_2_ plateau requirement in a ramp test does not mimic the original concept. In two of the four participants who did not achieve a VO_2_ plateau during the incremental test, true VO_2max_ had been achieved, while in the other two, the verification test yielded a higher VO_2max_. The secondary criteria for VO_2max_, which include the achievement of >85% of age-predicted maximum heart rate and an RER of >1.15, were present in three out of four participants who did not achieve a VO_2_ plateau and in two out of three participants who achieved their VO_2max_ during the verification test. Taken together, these results agree with findings from Poole et al. [30] and the subsequent recommendation of rejecting secondary criteria to validate VO_2max_ from incremental exercise tests.

The selected WR during the verification test must be sustainable for a sufficient duration such that VO_2_ kinetics allow for the achievement of VO_2max_ [18]. Wilkerson et al. [32] reported a decrease in test duration from 100% to 110% to 120% of VO_2max_ but did not report significant differences in VO_2_ (% peak) between these exercise sessions. In the present study, all participants achieved a VO_2_ plateau 80 s into the verification test. However, for two participants, verification VO_2_ was at least 2% lower than incremental VO_2_, which suggests that oxygen delivery or utilization was likely limited prior to achievement of VO_2max_ during the verification test in these participants.

Midgley et al. [28] reported that in four men who had a higher verification VO_2_ when compared with incremental VO_2_, all four had achieved a VO_2_ plateau during the incremental test. In the present study, of the three participants who had a higher verification VO_2_ when compared with incremental VO_2_, only one had achieved a VO_2_ plateau during the incremental test. It is apparent, from these limited findings, that the achievement of a VO_2_ plateau during the incremental test does not preclude a higher verification VO_2_. The three participants with a higher verification VO_2_ when compared with incremental VO_2_ in the present study exhibited a slightly longer verification test duration (142 ± 19 s) when compared with the eight participants without a higher verification VO_2_ (121 ± 19 s; *p* = 0.133; Mann–Whitney U test). A longer test duration could potentially explain the higher verification VO_2_. However, it is also possible that faster VO_2_ kinetics during the verification test as well as a priming effect from the previous incremental test [33] allowed for the attainment of VO_2max_ during the verification test but not during the incremental test for these three participants.

The average VO_2max_ in the current study of 88.8 ± 7.4% predicted is consistent with estimates of 89.7 ± 25.7% predicted from Jung et al. in 377 adults with prehypertension [34]. As noted in the introduction, quantifying VO_2max_ relative to body weight can significantly underestimate cardiorespiratory fitness in overweight and obese individuals, in whom metabolically inactive fat mass is unable to utilize oxygen during exercise [13,35]. It may be prudent to adopt the approach of quantifying cardiorespiratory fitness as a percent of predicted based on ideal body weight to get an unbiased estimate of cardiorespiratory fitness [13,14,15]. Although the current project was not a large epidemiological study, it does provide an accurate assessment of cardiorespiratory fitness that is not confounded by body mass in prehypertensive normal-weight and overweight men and women. These results are important for future work in the area of cardiorespiratory fitness, underlying comorbidities, and disease risk prediction.

This study had a small sample size and an uneven sex distribution, which precluded the analysis of sex differences. Furthermore, comparisons by overweight status were also not possible due to limited sample size. Finally, the AHA removed the term “prehypertension” towards the end of 2017. The new AHA guidelines classify systolic BP between 120 and 129 mm Hg and diastolic BP below 80 mm Hg as “elevated” BP and systolic BP between 130 and 139 mm Hg or diastolic BP between 80 and 89 mm Hg as stage 1 hypertension [36]. Based on this new definition, nine participants in this study would be classified with “elevated” BP and two with stage 1 hypertension. Despite these limitations, this study has several strengths, which include the confirmation of VO_2max_ using a verification test in adults with prehypertension, careful examination of individual test data for attainment of a VO_2_ plateau, and the quantification of VO_2max_ based on ideal body weight in normal-weight and overweight prehypertensive men and women.

## 5. Conclusions

Our data confirms that verification testing is feasible, validates the attainment of VO_2max_, and can potentially reduce the over diagnosis of functional impairment (i.e., deconditioning) in adults with prehypertension. Clinicians and researchers who routinely evaluate cardiorespiratory fitness in adults with prehypertension could consider verification testing as well as the quantification of cardiorespiratory fitness as a percent of predicted based on ideal body weight to achieve an unbiased and accurate estimate of cardiorespiratory fitness. This approach could help identify prehypertensive adults with low cardiorespiratory fitness who would benefit from exercise interventions to increase cardiorespiratory fitness and to reduce the risk of developing hypertension and cardiovascular disease.

## Figures and Tables

**Table 1:** Participant characteristics reported as mean ± *SD*.

Variable	All (*N* = 11)	Men (*N* = 7)	Women (*N* = 4)	*p*
Age (year)	22.5 ± 2.9	21.7 ± 2.4	23.8 ± 3.4	0.447
Height (cm)	170.4 ± 8.3	174.4 ± 5.5	163.4 ± 8.1	0.047
Weight (kg)	71.7 ± 12.6	77.0 ± 12.7	62.5 ± 5.5	0.059
BMI (kg·m^2^)	24.6 ± 3.2	25.2 ± 3.2	23.6 ± 3.5	0.450
Normal weight/Overweight	6/5	3/4	3/1	0.545
Resting HR (beats/min)	75 ± 7	73 ± 6	79 ± 8	0.155
Resting systolic BP (mm Hg)	125 ± 4	126 ± 3	124 ± 5	0.250
Resting diastolic BP (mm Hg)	75 ± 5	73 ± 3	79 ± 5	0.037
Maximum work rate (Watt)	206 ± 42	232 ± 24	160 ± 16	0.007

BMI: body mass index; HR: heart rate; BP: blood pressure.

**Table 2:** Individual data from the incremental (Incr) and verification (Ver) tests.

Sex	BMI (kg·m^−2^)	Incr VO_2_ (L·min^−1^)	Ver VO_2_ (L·min^−1^)	Ver VO_2_ > Incr VO_2_	Incr VO_2_ Plateau	Incr HR (% Pred)	Incr RER	Ver Duration (s)	VO_2max_ (% Pred)
F	20.6	1.74	1.73	No	No	98	1.39	96	94.53
F	21.9	1.67	1.74	No	No	93	1.42	103	88.49
F	23.3	1.43	1.64	Yes	No	97	1.44	122	89.69 *
F	28.5	1.54	1.54	No	Yes	84	1.47	121	84.17
M	20.7	2.22	2.12	No	Yes	89	1.33	139	76.62
M	22.3	2.67	2.82	Yes	Yes	91	1.28	107	97.77
M	22.8	2.77	2.79	No	Yes	95	1.37	115	98.26
M	26.8	2.46	2.51	No	Yes	91	1.54	150	87.07
M	27.2	2.64	2.57	No	Yes	89	1.39	145	85.62
M	27.6	2.95	2.93	No	Yes	96	1.42	157	96.24
M	29.1	2.46	2.71	Yes	No	79	1.15	137	78.56

F: female; M: male; BMI: body mass index; VO_2_: oxygen uptake; HR: heart rate; pred: predicted; RER: respiratory exchange ratio; max: maximum; * this participant would have been incorrectly classified as below normal for cardiorespiratory fitness if the verification test had not been completed.

**Table 3:** Measurements from the incremental and verification tests reported as mean ± *SD* (*N* = 11).

Variable	Incremental Test	Verification Test	*p*
VO_2_ (L·min^−1^)	2.23 ± 0.54	2.28 ± 0.54	0.213
VO_2_ (mL·min^−1^·kg^−1^)	31.56 ± 6.65	32.22 ± 6.26	0.213
VO_2_ (% predicted)	86.06 ± 8.52	88.13 ± 8.04	0.248
VCO_2_ (L·min^−1^)	3.07 ± 0.94	2.81 ± 0.77	0.091
RER	1.38 ± 0.10	1.21 ± 0.13	0.010
V_E_ (L·min^−1^)	87.47 ± 22.16	84.04 ± 23.20	0.374
HR (beats·min^−1^)	180 ± 11	180 ± 7	0.646
HR (% predicted)	91.04 ± 5.92	91.05 ± 3.24	0.646
V_T_ (L)	2.32 ± 0.63	2.33 ± 0.71	0.534
*f*_B_ (breaths·min^−1^)	38 ± 7	37 ± 9	0.477
RPE *	17.2 ± 1.3	17.0 ± 1.5	0.414
RPB *	5.5 ± 1.8	5.3 ± 2.1	0.705

VO_2_: oxygen uptake; VCO_2_: carbon dioxide production; RER: respiratory exchange ratio; V_E_: ventilation; HR: heart rate; V_T_: tidal volume; *f*_B_: breathing frequency; RPE: ratings of perceived exertion; RPB: ratings of perceived breathlessness; * *N* = 7 because RPB and RPE were not collected during the verification test for four participants.

## Data Availability

The data presented in this study are available on request from the corresponding author.
